# Localized efficacy of environmental RNAi in *Tetranychus urticae*

**DOI:** 10.1038/s41598-022-19231-3

**Published:** 2022-08-30

**Authors:** Nicolas Bensoussan, Maja Milojevic, Kristie Bruinsma, Sameer Dixit, Sean Pham, Vinayak Singh, Vladimir Zhurov, Miodrag Grbić, Vojislava Grbić

**Affiliations:** 1 Department of Biology, The University of Western Ontario, 1151 Richmond Street, London, ON N6A 5B7 Canada; 2 Present Address: Institut national de recherche pour l’agriculture, l’alimentation et l’environnement, 33882 Villenave d’Ornon, France; 3 Present Address: National Institute of Plant Genome Research, New Delhi, 110067 India

**Keywords:** RNAi, Entomology, Cellular imaging, RNAi, Histology, Fluorescence imaging, Molecular imaging

## Abstract

Environmental RNAi has been developed as a tool for reverse genetics studies and is an emerging pest control strategy. The ability of environmental RNAi to efficiently down-regulate the expression of endogenous gene targets assumes efficient uptake of dsRNA and its processing. In addition, its efficiency can be augmented by the systemic spread of RNAi signals. Environmental RNAi is now a well-established tool for the manipulation of gene expression in the chelicerate acari, including the two-spotted spider mite, *Tetranychus urticae*. Here, we focused on eight single and ubiquitously-expressed genes encoding proteins with essential cellular functions. Application of dsRNAs that specifically target these genes led to whole mite body phenotypes—dark or spotless. These phenotypes were associated with a significant reduction of target gene expression, ranging from 20 to 50%, when assessed at the whole mite level. Histological analysis of mites treated with orally-delivered dsRNAs was used to investigate the spatial range of the effectiveness of environmental RNAi. Although macroscopic changes led to two groups of body phenotypes, silencing of target genes was associated with the distinct cellular phenotypes. We show that regardless of the target gene tested, cells that displayed histological changes were those that are in direct contact with the dsRNA-containing gut lumen, suggesting that the greatest efficiency of the orally-delivered dsRNAs is localized to gut tissues in *T. urticae*.

## Introduction

Environmental RNAi refers to the ability of exogenously supplied dsRNA to induce silencing of complementary transcripts in cells of the recipient organism. In multicellular organisms, environmental RNAi involves the uptake of dsRNA by the primary groups of cells that may be followed by the spread of gene silencing into secondary groups of cells and tissues, leading to the systemic RNAi^1^. Since the discovery of RNAi in *Caenorhabditis elegans*^2^, environmental RNAi has been exploited as a tool to manipulate the expression of endogenous genes in many organisms. Efficient RNAi protocols, coupled with the availability of whole-genome sequences, enabled functional genomics studies^3–13^ bypassing a need for the generation of stable mutant alleles and maintenance of stocks of mutant lines. In addition to the utilization of RNAi as a reverse genetics tool, it is also being exploited in pest control. Although feeding, microinjection, and transgene expression can be used as delivery methods for RNAi experiments in functional genomics studies, pest control strategies are reliant on the oral delivery of RNAi-inducing RNAs. The initial demonstration of RNAi in crop protection has been based on the creation of transgenic plants expressing constructs leading to the generation of small RNAs against essential genes in the pest herbivore^14,15^. Subsequently, the demonstration that topically applied dsRNAs can lead to strong phenotypic responses in herbivorous pests^16^ opened the possibility of developing RNAi-based biopesticides. The ability of dsRNA to specifically target pest species through a mode of action that is independent of those associated with currently used pesticides makes RNAi-based biopesticides an attractive alternative to chemical-based control measures and an important tool for pesticide resistance management.

The utility of environmental RNAi depends on the stability of delivered dsRNA, its successful cellular uptake, and its ability to initiate target gene silencing. In some organisms, like Drosophila, RNAi acts cell-autonomously leading to gene silencing only in cells that generate dsRNA or that are direct recipients of the environmental dsRNA^17^. In others, like *C. elegans* and Coleopteran insects, locally initiated RNAi can spread to distant cells leading to systemic RNAi (reviewed in Joga et al.^18^). In such cases, the knockdown of target gene expression is typically greater than 90% when assessed on the whole-body sample^14,18–21^. In the absence/ineffective systemic RNAi, the RNAi-induced reduction of the target gene expression is low when assessed on the whole-body sample and is localized to cells that are the primary recipients of dsRNAs. For orally delivered environmental dsRNA, the localized RNAi effects are expected in the midgut cells where dsRNA uptake occurs. In *C. elegans*, SID-2-dependent endocytosis is required for the uptake of dsRNA by the intestinal cells^22,23^ and SID-1 enables the movement of dsRNA from cell to cell^1,23^. Systemic RNAi in *C. elegans* is further supported by the amplification of the RNAi signal through the generation of secondary siRNAs by the RNA-dependent RNA polymerase (RdRP)^24,25^. The endocytic pathway also mediates dsRNA uptake in insects^26,27^, however, it appears to be independent of SID-2, as *Sid-2* genes are absent in insect genomes analysed so far^28–31^. The SID-1, associated with systemic RNAi, is present in most insects but has not been identified in Diptera (reviewed in Joga et al.^18^). Congruently, systemic RNAi does not occur in Drosophila. Instead, in Drosophila, RNAi is limited to cells that are the primary recipients of dsRNA, leading to cell-autonomous RNAi phenotypes^17^. Furthermore, insects lack RNA-dependent RNA polymerase^28^. The mechanism of the amplification of the RNAi effect in insects is still unknown.

The two-spotted spider mite, *Tetranychus urticae*, is a herbivorous acari with an extremely wide host range that includes all major crops^32^. Its efficient xenobiotic responsiveness is also reflected in its ability to rapidly develop (multi)resistance to chemical pesticides used for mite control^33^. Driven by its importance as an agricultural pest, small genome size, and easy maintenance under laboratory conditions, *T. urticae* has been developed as a model chelicerate. The high-quality genome sequence of *T. urticae* is available^34^, establishing the basis for functional genomic studies. The analysis of genomic sequences revealed the presence of gene complements required for the processing of siRNA, miRNA, and piwi RNAs^34^, and mite sensitivity to RNAi was first demonstrated through the injection of double-stranded and small interfering RNAs^35^. Subsequently, various protocols for oral delivery of dsRNAs were developed^36^. A limited screen of mite homologues of *Tribolium castaneum* RNAi-sensitive targets revealed that oral delivery of dsRNA can dramatically affect mite mortality and fecundity^37^, opening a possibility for the development of RNAi-based mite pest control.

SID-like genes were not identified in the *T. urticae* genome, but similar to plants and the nematode *C. elegans*^38^*, T. urticae* contains multiple copies of RdRP encoding genes^34^. Consistent with the possibility that RdRP amplifies RNAi signal in *T. urticae*, transitive small RNAs (antisense transcripts mapping outside the target region within the target mRNA) were identified upon oral delivery of dsRNA^48^. The amplification of RNAi signal, the strength of the RNAi-induced phenotypes^37^, and the ability of maternally-injected RNAs to induce loss-of-function phenotype in the progeny^35^, suggest the highly efficient RNAi in mites. However, the modest knockdown of target genes with broad expression patterns when assessed at the whole mite level^37,39,40^ raises the question of the spatial reach of the environmental dsRNA. As the knowledge of cell types/tissues that are susceptible to the environmental RNAi will affect its use as a reverse genetics tool and is the main determinant of gene targets that can be used for the development of RNAi-based biopesticides, it is essential to map the spatial efficacy of orally-delivered dsRNAs. Here, we assessed the spread of RNAi triggered by the orally-delivered dsRNAs against genes predicted to be constitutively expressed and required for essential cellular functions. Our results indicate that the strongest RNAi effects are localized to cells that are the primary recipients of the environmental dsRNA in the gut tissue, indicating that environmental RNAi has localized efficacy in *T. urticae.*

## Results

### The effect of RNAi on the transcript abundance of target genes

**Figure 1:**
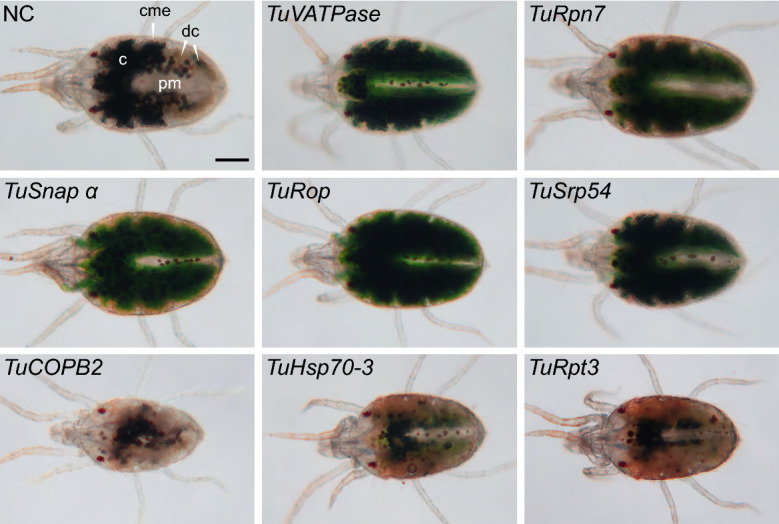
Mite body phenotypes upon dsRNA treatment. Two-spotted spider mites, *T. urticae*, are characterised by the presence of two dark spots, see NC, when observed under bright field. Spots are formed by the accumulation of dark digestive cells (dc) at the anterior of the midgut caeca (c) that can be seen through the semi-transparent cutile. Mites treated with dsRNAs targeting *TuVATPase*, *TuRpn7, TuSnap α*, *TuRop* and *TuSrp54* have a dark body phenotype characterized by the accumulation of a dark green pigment in the caeca lumen. Mites treated with dsRNAs targeting *TuCOPB2, TuHsc70-3* and *TuRpt3* displayed a spotless phenotype, characterized by the absence of the accumulation of black digestive cells. caeca (c); caecal midgut epithelium (cme); posterior midgut (pm); digestive cells (dc). Scale bar: 100 μm.

**Figure 2:**
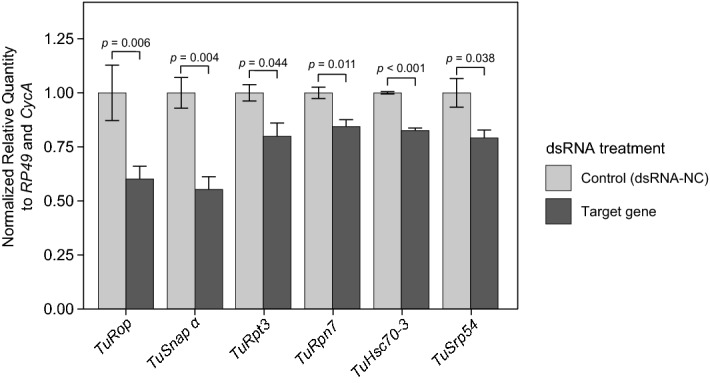
Target gene knockdown after dsRNA treatment. Average gene expression level relative to the expression of the reference control genes *RP49* and *CycA*. Data represent the mean ± SE. The RT-qPCR analysis was assessed at the whole mite level and was conducted in three independent experimental runs. Statistical analysis was performed using unpaired two-tailed *t* test (exact *P*-values corresponding to each pairwise comparison between the control and the treatment are displayed).

The two-spotted spider mite is characterized by its two prominent dark spots (Fig. 1-NC), that arise due to the see-through semi-transparent cuticle and reflect the presence of dark digestive cells at the anterior of the midgut caeca (dc and c respectively in Fig. 1). We have previously identified conservation in the sensitivity of RNAi targets between *T. urticae* and *Tribolium castaneum*^37^ (Table 1). The application of dsRNAs against these eight *T. urticae* target genes resulted in the visible body phenotypes (Fig. 1). Specifically, silencing of *TuRpn7*, *TuSnap α, TuRop,* and *TuSrp54* gave rise to a dark-body phenotype previously observed in dsRNA-*TuVATPase* treated mites^41^, while silencing of *TuHsc70-3* and *TuRpt3* resulted in a spotless phenotype also seen in mites treated with dsRNA-*TuCOPB2*^37^. As these body phenotypes were not observed in mites treated with dsRNA-NC and were developed in > 95% of mites treated with two independent dsRNA targeting each of the eight mite genes^37,41^, we concluded that these phenotypes result from the application of environmental dsRNA. To determine the effects of dsRNAs on the expression of their corresponding target genes, we designed gene-specific primer pairs for RT-qPCR analysis that are within the coding region and where at least one primer is outside of the sequences complementary to dsRNA fragments (Supplemental Table 2). Such arrangement ensures the analysis of the expression of the RNAi-targeted transcript and avoids cDNA contamination that results from the reverse transcription of dsRNA. Mite samples were collected at times when RNAi-associated phenotypic changes became visible but before the onset of mortality in the treated populations^37^, securing that target gene expression has been affected by the RNAi treatment. The expression of all target transcripts was significantly lower relative to the control treatment (Fig. 2 and Suzuki et al.^41^; Bensoussan et al.^37^). All tested genes are single genes in *T. urticae* genome. None dsRNA (except one targeting *TuRpt3*) had identity over any consecutive 21 bps to the sequence within *T. urticae* genome outside the target gene. dsRNA against *TuRpt3* has short stretches of sequence identity also present in *tetur35g00760* and *tetur02g06210* (Supplemental Fig. 1), however, the application of dsRNA-*TuRpt3* did not affect the expression levels of these potential off-targets. So, the mite body phenotypes are specific for the application of dsRNAs and are associated with downregulation of target gene expression ranging from 20 to 50%.

**Table 1:** List of *Tetranychus urticae* genes identified as highly efficient RNAi targets and used in this study.

Gene name	Tetur ID	Gene symbol	Cellular function
*Ras opposite*	*tetur13g00570*	*TuRop*	SNARE binding
*Alpha soluble NSF attachment protein*	*tetur06g05400*	*TuSnap α*	Vesicular transport
*Regulatory particle triple-A ATPase 3*	*tetur32g01800*	*TuRpt3*	Subunit of the 26S proteasome
*Regulatory particle non-ATPase 7*	*tetur33g01390*	*TuRpn7*	Subunit of the 26S proteasome
*Heat shock 70-kDa protein cognate 3*	*tetur08g01320*	*TuHsc70-3*	Protein folding
*Signal recognition particle protein 54k*	*tetur17g03110*	*TuSrp54*	Protein secretion
*Coatomer subunit beta 2*	*tetur24g00150*	*TuCOPB2*	Vesicular trafficking
*Vacuolar-type H*^+^*-ATPase*	*tetur09g04140*	*TuVATPase*	Proton pump, regulation of pH

### Localization of the dsRNA upon ingestion

**Figure 3:**
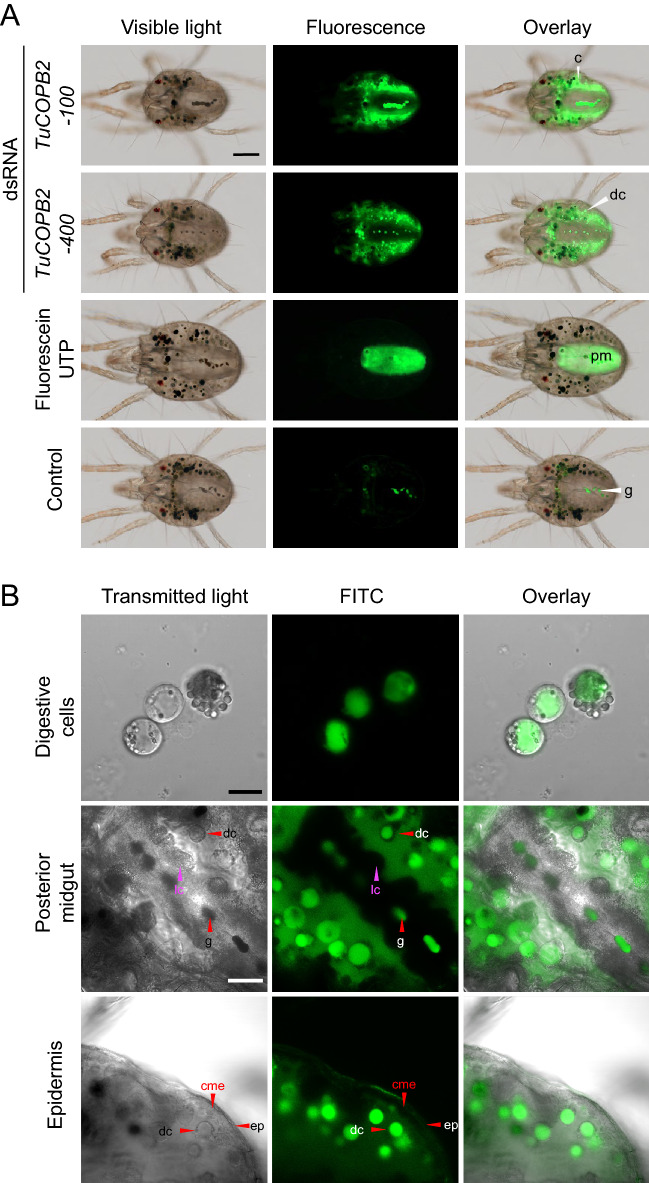
Localization of the ingested fluorescently labelled dsRNAs-*TuCOPB2*-100 and dsRNAs-*TuCOPB2*-400 following 24 h of mite feeding. (**A**) Fluorescence of labeled dsRNAs is seen in the mite caeca lumen (c) and digestive cells (dc); free fluorescein-12-UTP localizes in the posterior midgut (pm); guanine pellets (g), located in the posterior midgut, exhibit autofluorescence. (**B**) A close-up of fluorescently labelled dsRNAs in digestive cells, posterior midgut, and epidermis. caecal midgut epithelium (cme); large cell (lc); epidermis (ep). Scale bar: (A) 100 μm; (B) 10 μm for the digestive cells panels and 25 μm for the posterior midgut and epidermis panels.

One possibility for the modest knockdown of target genes is localized effectiveness of RNAi to cells that are the primary recipients of dsRNAs. To assess this possibility, we first determined the localization of the ingested dsRNAs. We fed mites with fluorescein-12-UTP-labeled dsRNAs *TuCOPB2*-100 and *TuCOPB2*-400 of 100 and 400 bp in length, respectively, and visualized their localization under a fluorescent microscope 24 h later. As seen in Fig. 3A, the fluorescein-12-UTP labeled dsRNAs were restricted to the caeca lumen and digestive cells, while free fluorescein-12-UTP was exclusively localized to the posterior midgut. Such separation of molecules based on their size is consistent with the previously reported size-exclusion filtering between the caeca and the posterior midgut^42^. Importantly, dsRNA-associated fluorescence does not appear in the posterior midgut, suggesting that dsRNA integrity may be retained throughout the first 24-h period. The retention and gradual accumulation of dsRNA in caeca as mites continuously uptake dsRNA indicate that cells that are lining the caeca are exposed to the highest concentration of dsRNA. However, dsRNA-associated fluorescence cannot be detected in midgut epithelial cells, nor in the cytoplasm of digestive cells (Fig. 3B).

### Expression patterns of RNAi-targeted genes

**Figure 4:**
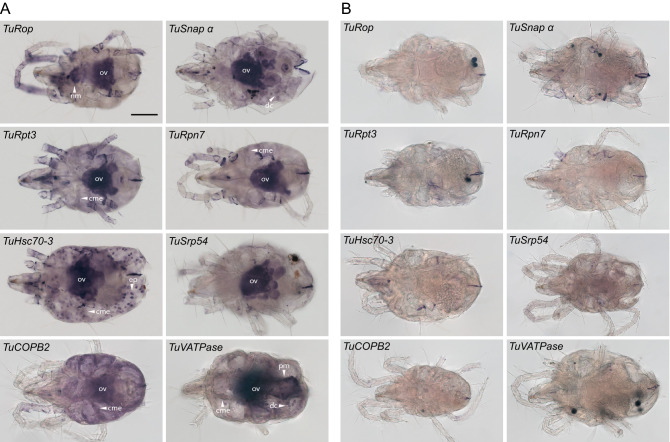
Whole-mount in situ hybridization of *TuRop, TuSnap α*, *TuRpt3*, *TuRpn7, TuHsc70-3, TuSrp54, TuCOPB2* and *TuVATPase* in adult *T. urticae* females. Anti-sense (**A**) and sense (**B**) digoxigenin-labeled probes. Description of the expression domains are presented in Supplemental Table 3. caecal midgut epithelium (cme); digestive cells (dc); posterior midgut (pm); nervous mass (nm); ovaries (ov); epithelium (ep). Scale bar: 100 μm.

Genes targeted here by RNAi are single genes in *T. urticae* that encode proteins with essential cellular functions (Table 1). Their *Tribolium* homologs are ubiquitously expressed, as assessed by tissue-specific RNASeq analysis, with enriched expression in some tissues (Supplemental Table 3). The expression of Drosophila homologs was assessed by the in situ hybridization, leading to the identification of the strongest and distinct expression domains (Supplemental Table 3). To determine the expression pattern of gene targets in adult female *T. urticae* mites we used digoxigenin-labeled antisense and sense RNA-probes unique to the target genes (Supplemental Table 2). Representative images of the whole-mount in situ stainings are shown in Fig. 4. Similar to the expression patterns in *Tribolium* and Drosophila, the pattern of target gene expression domains was not uniform. Invariably, target genes had the strongest signal in the ovaries (ov, Fig. 4A). In addition, some genes had strong signals in caecal midgut epithelial cells (cme, *TuRpt3, TuRpn7, TuHsc70-3, TuCOPB2,* and *TuVATPase*), digestive cells (dc, *TuSnap a* and *TuVATPase*), posterior midgut epithelial cells (pm, *TuVATPase*), epithelium (ep, *TuHsc70-3*) and neural mass (nm, *TuRop*) (Fig. 4A and Supplemental Table 3), while no signal was detected when sense RNA-probes were used (Fig. 4B).

### Histological analysis of midgut epithelial cells upon RNAi treatments

**Figure 5:**
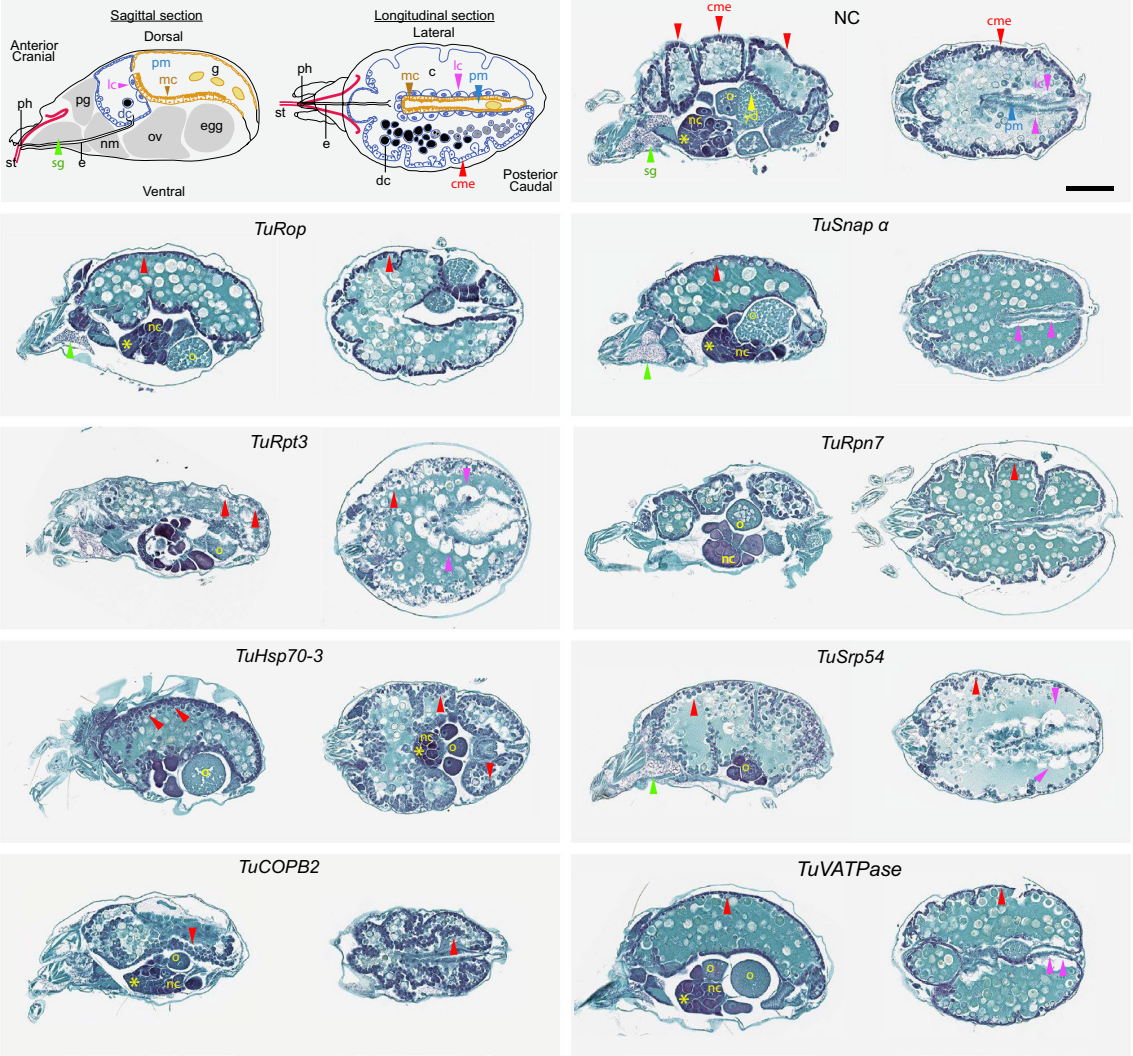
Histological analysis of mite RNAi phenotypes. Schematics depicting the internal anatomy of *T. urticae* female (top left, adapted from Bensoussan et al.^42^). On the left side of the panel—sagittal, and on the right—longitudinal sections through mite body. Stylet (st); pharynx (ph); esophagus (e); caeca (c); digestive cell (dc); microvilli cells in posterior midgut (mc in pm); prosomal gland (pg); nervous mass (nm); guanine pellet (g). Tissues of interest are labeled with arrowheads: red, caecal midgut epitheli cells (cme); magenta, large cells (lc); blue, posterior midgut (pm); green, silk gland (sg); yellow, ovaries: asterisk, previtellogenic cells; *nc* nurse cells; *o* oocyte; *yd* yolk droplets within the oocyte. Higher magnifications of tissues with perturbed cellular morphologies are shown in Fig. 6. Scale bar: 100 μm.

**Figure 6:**
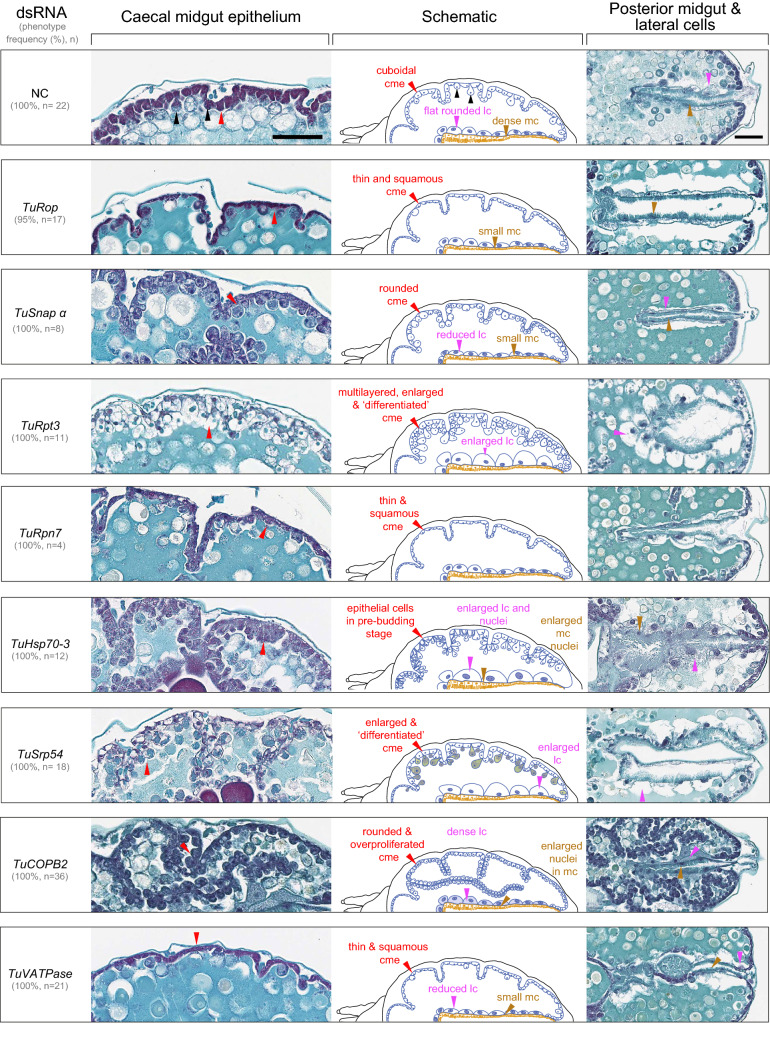
Histological phenotypes of midgut epithelial cells upon dsRNA treatments. Left, details of caecal midgut epithelial cells; right, details of large and posterior midgut cells in mites treated with dsRNAs; middle, schematics illustrating changes of midgut epithelial cells upon dsRNA treatments. Caecal midgut epithelial cells normally consist of cuboidal and densely stained cells (red arrowhead), rounded cells and enlarged cells that protrude into the caecal lumen (black arrowheads). magenta arrowhead, large cells (lc); brown arrowhead, microvilli cells in the posterior midgut. Scale bar: 50 μm.

As RNAi target genes are predicted to carry essential cellular functions, including membrane transport (*TuRop, TuSnap α*, *TuCOPB2*), 26S proteasome degradation (*TuRpt3* and *TuRpn7*), protein folding and secretion (*TuHsc70-3* and *TuSrp54),* and regulation of pH (*TuVATPase*) (Table 1), we reasoned that silencing these essential genes may lead to cellular phenotypes. Since these genes are ubiquitously expressed, patterns of perturbed cellular phenotypes upon oral delivery of dsRNAs targeting these genes could reveal tissue domains with high RNAi efficiency. To identify potential cellular phenotypes caused by the dsRNA treatments, we performed histological analysis of mite tissues at times when body phenotypes were apparent but prior to the onset of mite mortality. Although macroscopic changes led to two groups of body phenotypes (dark and spotless, Fig. 1), silencing of target genes was associated with the distinct cellular phenotypes (see representative images of mite histological sections in Figs. 5 and 6). Our previous analysis of the mite digestive track identified three major types of gut epithelial cells^42^. The midgut epithelium, a single-cell layer, is divided into caecal midgut epithelium (cme, red arrowheads in Fig. 5) that constitutes the outer midgut wall, large cells (lc, magenta arrowheads in Figs. 5 and 6) that form an inner wall separating caeca and the posterior midgut and the posterior midgut that runs posteriorly along the dorsal midline (pm, blue arrowhead in Fig. 5). The lateral walls of the posterior midgut are made of epithelial cells with microvilli (mc, Fig. 5).

While the majority of caecal midgut epithelial cells normally consist of cuboidal and densely stained cells **(**red arrowhead, NC in Fig. 6), there are some rounded cells and enlarged cells that protrude into the caecal lumen (black arrowheads, NC in Fig. 6). Latter cells ultimately bud off to give rise to digestive cells that are free-floating in the ceacal lumen. The appearance of the caecal midgut epithelium cells was affected by the application of dsRNAs. They are: (a) thin and squamous in mites treated with dsRNA targeting *TuRop, TuRpn7* and *TuVATPase*; (b) enlarged, and appear differentiated in mites with silenced *TuRpt3* and *TuSrp54*; (c) rounded in dsRNA-*TuSnap α* and dsRNA-*TuCOBP2* treated mites; (d) overproliferated in dsRNA-*TuCOBP2* treated mites; and (e) attached to the basement membrane but are enlarged and budding into the caecal lumen, in mites treated with dsRNA-*TuHsc70-3* (Figs. 5 and 6). Changes in the histological appearance of large cells are also observed (Figs. 5 and 6). For example, these cells are enlarged in mites treated with dsRNA against *TuRpt3, TuHsc70-3* and *TuSrp54*, with enlarged nuclei in dsRNA-*TuHsc70-3* treated mites, reduced in size in mites treated with dsRNAs targeting *TuVATPase* and *TuSnap α,* and differentially stained in dsRNA-*TuCOBP2* treated mites. Changes of cells with microvilli in the posterior midgut are less pronounced but include enlarged nuclei in dsRNA-*TuHsc70-3* treated mites and reduced size in mites treated with dsRNA against *TuVATPase, TuSnap α,* and *TuRop* (Figs. 5 and 6)*.*

### The analysis of RNAi treatments on digestive cells

**Figure 7:**
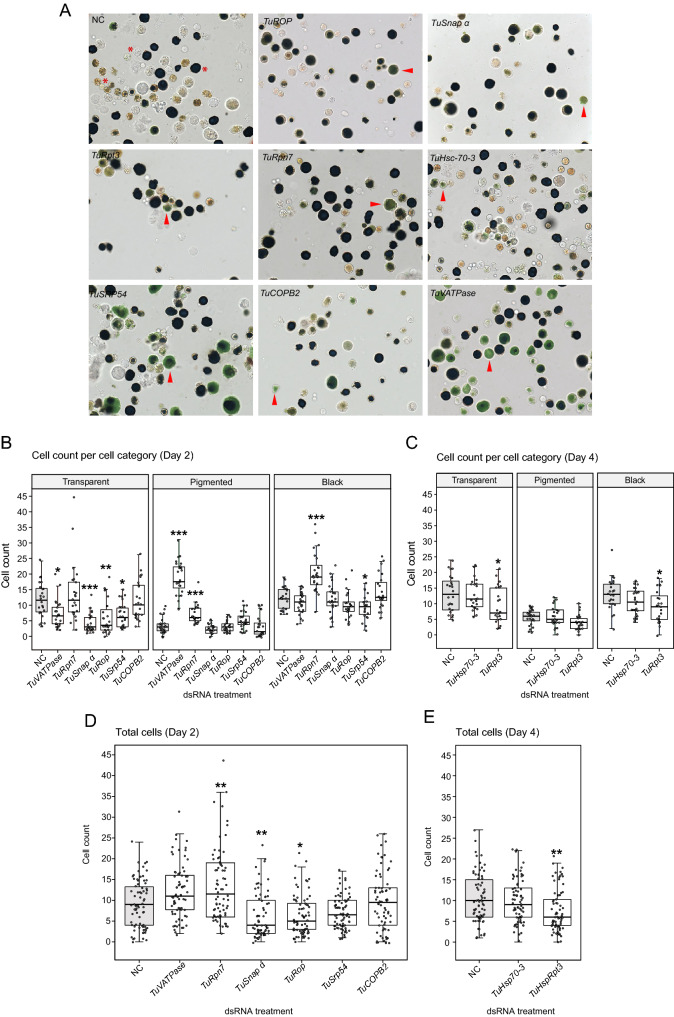
The effect of dsRNA treatments on digestive cells. (**A**) Representative images of digestive cells dissected from RNAi-treated mites. Digestive cells undergo maturation from transparent, pigmented to black, see cells labeled with asterisk in NC panel. (**B**,**C**) Cell count per cell type category: transparent, pigmented, and black at 2 days (**B**) and 4 days (**C**) post RNAi treatment. (**D**,**E**) Total cell counts at 2 days (**D**) and 4 days (**E**) post RNAi treatment. The box indicates the first and the third quartile, the middle line represents the median, and the whiskers indicate the 1.5 interquartile range. Individual data points are plotted as grey circles. Statistical analysis was performed using a negative binomial regression followed by posthoc pairwise comparisons between estimated marginal means of each RNAi treatment against the control group with subsequent *P*-value adjustment following the Bonferroni method (not significant, *P* > 0.05; *P < 0.05; **P < 0.01; ***P < 0.001).

A dark body phenotype is characteristic for mites treated with dsRNAs against *TuVATPase, TuRpn7*, *TuSnap α, TuRop,* and *TuSrp54* (Fig. 1). It results from the dark green color of the caecal lumen. As mites feed from the content of mesophyll cells that contain chlorophyll, the lumen color likely reflects the accumulation of ingested plant cell content, indicating changes in the mite’s digestive physiology. In mites, the digestion of plant material has been proposed to occur in digestive cells that bud off of the midgut epithelia and are free-floating in the caecal lumen (Fig. 5 and Bensoussan et al.^42^). Newly released digestive cells are initially transparent, but as they internalize the gut lumen, pigmented vesicles become apparent. Finally, digestive cells accumulate waste products of digestion^42–44^ that appear as dark deposits in a single large vesicle. We refer to digestive cells in these physiological states as transparent, pigmented, and black, respectively (marked with red asterisks in Fig. 7A). To determine if silencing of RNAi targets that result in mite body phenotypes perturbed digestive cells' physiology, we dissected digestive cells from the caecal lumen of dsRNA-treated mites and examined their appearance under a microscope. While pigmented cells normally appear brownish, almost all pigmented digestive cells in mites treated with dsRNAs against *TuVATPase* and *TuSrp54* prominently show a novel ‘green’ phenotype (arrowheads in Fig. 7A). Sporadic accumulation of green pigment in digestive cells is also seen upon silencing of *TuRpn7, TuCOPB2, TuHsc70-3, TuSnap a*, and *TuRpt3* (Fig. 7A)*.* Another qualitative effect of RNAi on digestive cells is seen in mites treated with dsRNA-*TuRpt3*, where cells form clumps upon dissection. In addition, an increased density of the caeca lumen was observed in mites with dark body phenotypes. The highest density was seen in mites treated with dsRNA against *TuSnap α, TuRop,* and *TuSrp54,* intermediary density in dsRNA-*TuRpn7* treated mites, and the lowest but still above the normal for dsRNA-*TuVATPase* treated mites. Furthermore, we determined quantitative differences for each cell type category (Fig. 7B,C) and total numbers of digestive cells (Fig. 7D,E) in dissected mite samples. Interestingly, among dark body phenotypes, only RNAi treatment against *TuVATPase* and *TuRpn7* showed a significant increase of pigmented cell numbers compared to the control dsRNA-NC treated mites (Fig. 7B). The majority of these cells displayed a novel ‘green’ phenotype (Fig. 7A). Thus, our data suggest that the dark body phenotype can arise either through the increased number of ‘green’ digestive cells (potentially impaired in the digestion process) or the accumulation of plant ingested material in the caeca lumen (in this case, digestive cells might be impaired in the acquisition of materials from the lumen). Among spotless phenotypes, only mites treated with dsRNA against *TuRpt3* displayed a significant reduction in transparent and black cell numbers compared to their corresponding controls (Fig. 7B,C). When total cell number is considered, a significant increase in digestive cell numbers was observed in *TuRpn7* silenced mites with about 50% more cells than in the control-treated mites, while a significant reduction in the number of digestive cells was observed in mites treated with dsRNAs targeting *TuRop*, *TuSnap α*, and *TuRpt3* with a decrease of about 30%, 32%, and 29% of cells compared to the control-treated mites (Fig. 7D,E).

### The effects of RNAi treatments on non-digestive tissues

To determine the effects of dsRNA treatments on non-digestive tissues we followed histological phenotypes associated with ovaries and silk glands cells, which are in immediate proximity to ventral caecal midgut epithelial cells. Ovaries were of particular interest, as silencing of most of the RNAi gene targets resulted in reduced mite fecundity^37^ that may have resulted from RNAi-induced histological changes in ovarian cells. Ovaries, situated in the ventral region of the mite body, have several cell types including the germ region and previtellogenic oocytes that are located most anteriorly (marked by the asterisk in Fig. 5). At their posterior, these cells are surrounded by three-nucleated nurse cells (nc, Fig. 5), further followed posteriorly by the oocytes at different maturations states—reflected in the abundance of yolk droplets (o and yd, respectively; Fig. 5 and Mothes, U. & Seitz, K. A.^71^). Silk glands (sg) are paired cells (one at each side of the esophagus) whose cytoplasm is filled with secretory vesicles (Fig. 5). In all dsRNA treatments, ovarian cell types had a normal histological appearance. Likewise, the density and the size of the secretory vesicles in silk glands of dsRNA-treated mites appear indistinguishable from those in mites treated with dsRNA-NC. Therefore, at the level of our histological analysis, ovarian and silk gland cells do not appear to be affected by the application of dsRNAs complementary to mite orthologues of *Tribolium*-sensitive RNAi targets (Fig. 5).

## Discussion

The reduction of target gene expression upon the application of the environmental dsRNA is typically used as experimental evidence for the effectiveness of RNAi. In many insects, 70–90% of transcript knockdown of constitutively-expressed genes is common^14,19–21,45–47^. However, silencing mite homologs of ubiquitously-expressed *Tribolium* sensitive RNAi targets led to partial transcript knockdown, ranging from 20 to 50%, when assessed at the whole mite level (Figs. 2 and 4). This is consistent with levels of target gene downregulation obtained in other RNAi studies in *T. urticae*^39–41,48–53^. The partial reduction of target gene expression is frequently associated with the overall low effectiveness of RNAi^54–57^. However, in mites, the partial gene knockdown is associated with strong RNAi-associated phenotypes (mite body phenotype (Fig. 1) and reduced mite fitness—survivorship and fecundity^37^). The dsRNA instability is an unlikely contributor to incongruency between the strong phenotypic effects of RNAi and the modest levels of target gene knockdowns since fluorescence associated with the fluorescein-12-UTP-labeled dsRNAs accumulated at high concentrations in mite caecal lumen while their breakdown products were undetectable in the posterior midgut (Fig. 3). In addition, the ability of injected dsRNAs to trigger phenotypic changes in the injected mites or their progeny indicates that mite cellular environments do not destabilize dsRNA or siRNAs^35,53^. An alternative possibility that can explain strong RNAi phenotypes despite the low level of target knockdown is RNAi-induced translational inhibition of the targeted transcripts rather than mRNA cleavage^58,59^. But, if that was the case, the RNAi phenotypes resulting from silencing ubiquitously-expressed genes would be widespread, throughout the mite body. However, histological analysis of mite tissues indicated the greatest RNAi responsiveness in midgut epithelial and digestive cells (Figs. 5, 6, 7). The lack of visible RNAi effects in ovaries, tissues that are adjacent to the midgut, may have resulted from high expression levels of target genes (Fig. 4), however, highly-expressed ovarian targets such as vitellogenin and its receptor were successfully downregulated in ticks^60^ and the mite *Panonychus citri*^61^ through the injection and oral delivery of complementary dsRNAs, respectively. At present, we cannot distinguish between the localized effectiveness of RNAi from differential tissue sensitivity to RNAi. Despite the prevalent effects of RNAi on cells directly in contact with dsRNAs in caecal lumen, we have also observed RNAi-associated histological phenotypes in cells that are lining the posterior midgut, a compartment that has limited direct exposure to the environmental dsRNAs (Figs. 3, 5 and 6). In addition, silencing genes encoding charged multivesicular body proteins (CHMPs) in *T. cinnabarinus* (closely related to *T. urticae*^62^) through the oral application of dsRNAs led to changes in mite locomotion that presumably resulted from alterations in muscle and/or neuronal cells^53^. These phenotypes indicate that other mite tissues can be targeted with orally-delivered dsRNAs. Comparative analysis of the effectiveness of RNAi, triggered by dsRNAs targeting genes with well-defined expression domains that are delivered orally and by injection, could discriminate between the localized effectiveness of RNAi and differential tissue sensitivity to RNAi in *T. urticae.*

Contrary to the localized effectiveness of RNAi in *T. urticae,* the highly significant effects of RNAi on target gene expression was seen in other mite species, including herbivorous *Panonicus citri*^61,63^, predatory *Metaseiulus occidentalis*^64,65^ and *Varroa destructor*^66,67^ mites and ticks^60^. The species-specific differences that result in localized RNAi effects in *T. urticae* are not known. The difference may be at any step in the RNAi process, namely: (a) the uptake of dsRNA from the gut lumen; (b) the dsRNA processivity in the cytoplasm in the primary recipient cell; (c) the amplification of the RNAi signal; and (d) the spread of the RNAi signal to other cells. The mechanism of dsRNA uptake in *T. urticae* is not known. SID-like genes were not identified in *T. urticae* genome^34^, but as in ticks, predatory mites and many insects, the uptake of the exogenous dsRNAs may occur via receptor-mediated endocytosis^31,60,64,68–70^. The class B scavenger receptor CD36 (SRB) is a surface receptor that has been shown to be crucial for the establishment of systemic RNAi in the tick *Haemaphysalis longicornis*^60^. The *T. urticae* genome has seven scavenger receptors and among them, *tetur13g00340* shares the highest identity with the ortholog of SRB from *H. longicornis* (accession number: KAH9373951). ‘RNAi-of-RNAi’ experiments could directly test the requirement of this and related scavenger receptors for dsRNA uptake in *T. urticae*. If dsRNA uptake in *T. urticae* occurs via endocytosis, then it differs from the process of the gut lumen uptake by digestive cells. Digestive cells of mites fed with the fluorescein-12-UTP-labeled dsRNAs accumulate fluorescence in the central vesicle/lysosome (Fig. 3B). However, these dsRNAs do not leave the central vesicle, a prerequisite for cytoplasmatic processing of dsRNA by the DICER complex, suggesting that vesicles containing the RNAi-promoting dsRNAs are different. We were unable to visualize vesicles containing fluorescein-12-UTP-labeled dsRNAs in midgut cells (Fig. 3B), even though they could have been seen in Drosophila S2 cells^68^ and in mite oocytes upon injection of dsRNAs^35^. The dsRNA cellular uptake seems to be efficient and widespread in *T. urticae* as orally-delivered dsRNAs are causing strong phenotypes in midgut cells (Figs. 5, 6, 7) and injected dsRNAs are inducing phenotypes in non-gut tissues^35,53^. If dsRNAs are imported via endocytosis, then their release from the endosome into the cytoplasm and processivity by the RNAi machinery seems to be efficient given the strong RNAi phenotypes in midgut cells (Figs. 5, 6, 7). As mites do not have hemolymph^42,71^, the spread of the RNAi signals is likely based on plasma membrane channels that allow cell-to-cell transfer of RNAi signals. The identities of these channels are not known, but in a range of organisms SID1(-like) proteins are required^72–74^. If differences in the RNAi efficiency between *T. urticae* and other acari are due to the efficiency of the spread of the RNAi signal, then comparative gene analysis of components of the export of RNAi signals could identify candidate genes whose subsequent analysis through ‘RNAi-of-RNAi’ experiments would establish their requirement for systemic RNAi.

Even though the histological changes were not visible in tissues outside the midgut we noted a strong reduction of fecundity in most RNAi-treated mites^37^. It remains unclear whether this phenotype is an indirect effect of altered digestion and consequently limited provision of nutrients to growing ovarian eggs or whether it reflects the reduced expression of target genes in the ovaries. Because of the high expression of target genes in ovaries, it is possible that the limited silencing of targets remains undetectable at the histological level. However, even limited downregulation of gene expression of a target may be a rate-limiting factor in oogenesis leading to reduced fecundity.

The localized effectiveness of orally delivered dsRNA has several implications on the utility of RNAi in *T. urticae*. First, it is expected to result in the partial transcript knockdown of ubiquitously-expressed genes when measured on the whole mite sample. As such, the analysis of the knockdown of such genes may not be a reliable predictor of the RNAi efficiency (Figs. 1 and 2). Second, as orally delivered dsRNA has reduced efficacy in non-gut tissues, RNAi experiments that target genes expressed outside the gut cells should use injection as a mode of dsRNA delivery. Third, since there are increased efforts to use RNAi toward the *T. urticae* pest control, the development of such pest management tools should focus on targeting genes that are essential in gut tissues. The last several years have seen the development of multiple RNAi protocols for *T. urticae*^35,40,41,53,75^. They supported the use of RNAi as a reverse genetics tool^49–53^ and the discovery of target candidate genes for sprayable RNAi pesticides^37^. A greater understanding of the global RNAi machinery will further increase the utility of RNAi in *T. urticae* and the range of its applications.

## Methods

### Mite rearing

*Tetranychus urticae* (London strain, Grbic et al.^34^) was maintained on bean plants (*Phaseolus vulgaris,* variety ‘California Red Kidney’; Stokes, Thorold, ON) grown in soil (Pro-Mix BX Mycorrhizae; Premier Tech, Rivie`redu-Loup, QC), in a controlled environment under 100–150 μmol m^−2^ s^−1^ cool-white fluorescent light, with a 16/8 h light/dark photoperiod and at a temperature of 26 °C.

### dsRNA synthesis and oral delivery

dsRNA fragments were synthesized using primers listed in Supplementary Table 1A as described in Bensoussan et al.^37^. Briefly, total RNA extraction and synthesis of cDNA was performed using the RNeasy Mini Kit (Qiagen, Valencia, CA) and the SuperScript II cDNA Synthesis Kit (Thermo Fisher Scientific, Waltham, MA), respectively, following manufacturers’ instruction. Templates for dsRNA synthesis were prepared by polymerase chain reaction (PCR) amplification using specific forward and reverse primers with a minimal T7 promoter sequence at their 5′ ends (see Supplemental Table 2). The template for the dsRNA-NC (382 bp) was prepared from genomic DNA as it has complementarity against the non-transcribed intergenic region 1,690,614–1,690,995 of the *T. urticae* genomic scaffold 12^34,41^. Amplified PCR fragments were purified with the Gel/PCR DNA Fragments Extraction Kit (Geneaid Biotech, New Taipei, Taiwan), denatured at 95 °C, and slowly cooled down to room temperature. dsRNA solutions were applied to newly molted adult female mites at concentrations of 500 ng/mL, following the soaking method described by Suzuki et al. ^36^.

### Imaging of mite body phenotypes

Images of mite body phenotypes following the dsRNA treatments were taken 2 days post soaking, except for mites treated with dsRNAs against *TuRpt3* and *TuHsc70-3* that were taken 4 days post dsRNA treatment. Images were taken using a stereomicroscope Leica MZ FLIII (Leica Microsystems, Wetzlar, Germany) fitted with the Canon EOS Rebel T5i camera (Canon, Japan). The experiment was conducted in three independent trials.

### RT-qPCR

For the target gene expression analysis post dsRNA treatments, ≥ 30 adult mites were collected at 2 days or 4 days for *TuRpt3* and *TuHsc70-3* post-treatment in three independent experimental trials. RNA was extracted using the RNeasy Mini Kit (Qiagen). Six hundred nanograms of RNA were reverse transcribed with the Maxima First Strand cDNA Synthesis Kit for RT-qPCR (Thermo Fisher Scientific). The RT-qPCR analysis was performed using forward and reverse primers that amplified non-overlapping fragments to those used for the synthesis of the dsRNAs (see Supplementary Table 2). Cycle threshold (Ct) values were averaged from three technical replicates. Expression values were normalized to the average of the reference genes *RP49* (*tetur18g03590*, coding for a ribosomal protein), and *CycA* (*tetur01g12670*, coding for the protein Cyclophilin A), and then to a mean of the control group treated with dsRNA-NC. Differences in the mean of normalized values between the control and treatments were analyzed with an unpaired two-tailed *t* test. Differences were considered significant at *P* < 0.05 for all analyses.

### In situ hybridization

Whole-mount in situ hybridization in *T. urticae* was performed as described by Dearden et al.^76^, with some modifications. Total RNA was extracted from *T. urticae* adult females as described above. PCR reactions using primers F-T7 with R were used to generate the sense template and primers using F and R-T7 were used to generate antisense templates for in vitro transcription. For generating the sense probe, a set of primers with only the forward primer containing the T7 promoter was used. For the antisense probe, only the set of primers with the reverse primer containing the T7 promoter was used. Depending on the primer combination, sense and antisense probes labeled with digoxigenin (DIG) were generated using T7 RNA polymerase (Roche, Anderlecht, Belgium) and DIG-UTPs (Roche) in the in vitro labeling reaction. The probes were then purified using SigmaSpin Sequencing Reaction Clean-Up Columns (Sigma) and mixed in a 1:1 volume of hybridization buffer (50% formamide (Sigma), 4 × SSC (Sigma), 1 × Denhardt’s solution (Sigma), 250 μg/mL heparin (sodium salt, Sigma), 0.1% Tween-20 (Sigma), 5% dextran sulfate (sodium salt, Sigma), and stored at − 20 °C. *T. urticae* mites were fixed using 4% formaldehyde in a solution of PBS with 1% Triton X-100 at RT for ~ 4 h and then at 4 °C for ~ 16 h. The formaldehyde solution was then replaced with PTw (PBS with 0.1% Tween-20) and the mites were briefly sonicated in an ultrasonic bath sonicator. Sonicated mites were re-fixed in 4% Formaldehyde in PTw for 1 h. The mites were then washed five times with PTw every five minutes and pre-hybridized in hybridization buffer (50% formamide, 1 × Denhardt’s solution, 250 μg/mL heparin (sodium salt), 0.1% Tween-20, and 5% dextran sulfate (sodium salt)) at 52 °C for 1 h. The mites were then incubated in a probe solution (10 μL of probe in 400 μL of hybridization buffer) overnight at 52 °C. Next, mites were washed three times for 30 min with wash buffer (50% formamide, 2 × SSC, 0.1% Tween-20) at 52 °C. The mites were washed for 20 min at RT with PTw, followed by two 20-min washes with PBTw blocking solution (1% BSA in PTw). The mites were then incubated overnight at 52 °C in a solution with a 1:1000 dilution of anti-digoxigenin antibodies conjugated with an alkaline phosphatase (AP) enzyme (Fab fragments, Roche) in PBTw. Unbound antibodies were removed by washing mites at RT with three 30-min PTw washes. The mites were washed twice with AP buffer (100 mM Tris pH 9.5, 100 mM NaCl, and 1 M MgCl_2_) for 10 min, and then stained in the dark with AP buffer containing 4.5 μL/mL of nitro blue tetrazolium (Sigma) and 3.5 μL of bromo-chloro-indolyl-phosphate (Sigma). After purple staining of the target was visible, the mites were washed in 100% methanol to reduce background staining and were then stored or mounted in 50% glycerol in PTw.

### Paraffin embedding and sectioning of mite tissues

RNAi-treated mites displaying body phenotypes were collected and fixed for 24 h at 4 °C in a solution of 4% formaldehyde in 10 mM phosphate buffer saline (PBS), pH 7.4 with Triton X-100 at 1% (v/v). Dehydration was done through a graded ethanol series (10%, 30%, 50%, and 70%; v/v in H_2_O). Mites were subsequently processed for paraffin infiltration and embedding in an automated tissue processor (Leica, ASP300TP). Tissue sections of 5 µm thickness were obtained with a microtome (Leica RM2255), dewaxed for 10 min in two baths of 100% xylene, and gradually rehydrated. Mite tissue sections were dyed using general staining with 0.1% Safranin O (C.I. 50240; MilliporeSigma) and 0.05% Fast Green (FCF, C.I. 42053, MilliporeSigma) solutions. Images were taken with a Zeiss AxioCam Color HRc CCD Camera 412-312 fitted on a Zeiss Axioplan II microscope.

### Fluorescent labelling of TuCOPB2 dsRNA

To visualize the dsRNA distribution in mite upon oral delivery, *TuCOPB2*-100 and *TuCOPB2*-400 were labelled with fluorescein-12-UTP according to the manufacturer’s protocol (Roche, catalog number 11427857910). Labelled dsRNAs were delivered to synchronized newly molted adult mites using the mesh delivery method described in Ghazy et al.^75^ for 24 h. Mites were mounted in 50% glycerol in PBS to be imaged under a fluorescent microscope. Fluorescence was visualized using an epifluorescence Zeiss Axioplan II microscope fitted with a FITC filter cube. Images were taken using an AxioCam Color HRc CCD Camera 412-312.

### Collection and analysis of digestive cells in dsRNA-treated mites

Synchronized adult female mites were treated with dsRNA using the soaking method as described above. Thirty adult female mites displaying phenotypes at either 2 days or 4 days post-treatment (for *TuRpt3* and *TuHsc70-3* targets), were collected in 0.5 mL tubes in a solution of 1X PBS with 0.1% Tween 20. A batch of ten mites was pipetted with a P10 and placed on a microscopy glass slide. To release digestive cells from the mite gut, a small perforation was made on a side of the lateral caeca with sharp point tungsten micro-dissecting needle (Roboz, USA, RS-6063) attached to a holder (Roboz, USA, RS-6060). Floating cells were collected from the glass slide and placed in 0.5 mL tubes and resuspended in a 1 × PBS and 0.1% Tween 20 solution to a final volume of 25 µL. Digestive cells in suspension were gently mixed by pipetting up and down and 5 µL was loaded in each hemocytometer chamber. A total of eight square surface areas (four squares from each corner of the two chambers of the hemocytometer) were used for each batch. To simplify the analysis, digestive cells were classified into three categories: transparent, pigmented, and black. The transparent cell category corresponds to the early developmental stage where the free-floating digestive cells are rounded and filled with transparent small vesicles (Fig. 7A). The pigmented cells are digestive cells whose internal vesicles display a coloration ranging from pale yellow to dark green (Fig. 7A). In the RNAi treatments targeting *TuVATPase* and *TuSRP54*, pigmented cells are filled with green vesicles. The black cells display dark brown or black internal vesicle(s) of different sizes. The cells were mounted in 50% (v/v) glycerol diluted in 1X PBS for taking the images shown in Fig. 7A. Mite digestive cell count data were analysed with a negative binomial regression model using the glm.nb function in the R package MASS (version 7.3.54). Residual diagnostics and interpretation generated from the model were inspected using the R package DHARMa (version 0.4.4.). Post-hoc pairwise comparisons between estimated marginal means of each RNAi treatment against the control group were performed using emmeans R package (version 1.7.2) with subsequent P-value adjustment following the Bonferroni method.

## Supplementary Information

Supplementary Information.

## Data Availability

The datasets generated and analysed during the current study are available in the BioStudies repository, https://www.ebi.ac.uk/biostudies/studies/S-BSST832?key=3635e448-917d-4210-9999-0743add47461.
