# Supplementary material for: Localized efficacy of environmental RNAi in Tetranychus urticae
Source: Sci Rep. 2022 Aug 30;12:14791. doi: 10.1038/s41598-022-19231-3 (PMC9427735; doi:10.1038/s41598-022-19231-3)
Supplement: Supplementary file 1 — Supplementary Information. [file 41598_2022_19231_MOESM1_ESM.pdf]

Localized efficacy of environmental RNAi in *Tetranychus urticae*

Authors: Nicolas Bensoussan[a,1], Maja Milojevic[a], Kristie Bruinsma[a], Sameer Dixit[a,2], Sean Pham[a], Vinayak Singh[a], Vladimir Zhurov[a], Miodrag Grbić[a], Vojislava Grbić[a,*]

[a]Department of Biology, The University of Western Ontario, 1151 Richmond Street, London, ON N6A 5B7, Canada.

[*]corresponding author

Current addresses: [1]Institut national de recherche pour l'agriculture, l'alimentation et l'environnement, 33882 Villenave d'Ornon, France; [2]National Institute of Plant Genome Research, New Delhi-110067, India.

(Ref: Submission ID f0c50b00-017e-4ab6-9273-b5cd0a7bffd6)

Additional information

Supplemental Table 1. Genes with consecutive 21 bp-sequence identity to dsRNA used for the RNAi.

Supplemental Table 2. Primers used in this study.

Supplemental Table 3. Expression patterns of RNAi-targeted genes.

Supplemental Figure 1. Expression analysis and pairwise sequence alignments of genes with consecutive 21 bp-sequence identity to dsRNA-*TuRpt3*.

Supplemental Table 1. Genes with consecutive 21 bp-sequence identity to dsRNA used for the RNAi

gene name	Tetur ID	# of off-targets (21 nt consecutive)	Sequences producing significant alignments	Length (nt)	Score (Bits)	E Value
TuRpt3	*tetur32g01800*	2	*tetur32g01800*	length:1248	1079	0
			tetur35g00760	length:1356	221	3.00E-57
			tetur02g06210	length:2394	84.2	5.00E-16
TuHsc70-3	*tetur08g01320*	0	*tetur08g01320*	length:1986	1025	0
TuRpn7	*tetur33g01390*	0	*tetur33g01390*	length:1170	1153	0
TuSnap	*tetur06g05400*	0	*tetur06g05400*	length:882	1139	0
TuSrp54	*tetur17g03110*	0	*tetur17g03110*	length:1494	1236	0
TuRop	*tetur13g00570*	0	*tetur13g00570*	length:1761	1187	0
TuCOPB2_100	*tetur24g00150*	0	*tetur24g00150*	length:2421	176	1.00E-44
TuCOPB2_400		0	*tetur24g00150*	length:2421	704	0
TuCOPB2_600		0	*tetur24g00150*	length:2421	1104	0
TuVATPase_600	*tetur09g04140*	0	*tetur09g04140*	length:1866	1133	0

Supplemental Table 2. Primers used in this study

Name	Application	Forward primer sequence (5'-3')	Reverse primer sequence (5'-3')	
	**RNAi**			**Fragment size**
TuSrp54	RNAi	[T7]-AGCAGTTACCAACTCTCCCA	[T7]-AGCAGCCCCCTGTAATTGTC	685bp
TuRop	RNAi	[T7]-CGAGGCTCCAGAAAAACAC	[T7]-GATGCACTTATCTGCAGGCG	658bp
TuSnap-α	RNAi	[T7]-GATTGTTCGGGGGCTCTTCT	[T7]-TCCCGGGAATCACTGAAAGC	631bp
TuHsc70-3	RNAi	[T7]-AACCTACAGCTGCTGCCATT	[T7]-GGGTTAATGCCCCGAGTAGG	568bp
TuRpn7	RNAi	[T7]-AACTGCTGGAGCGTATGAGG	[T7]-AATCTTCGACCCACGCAAGT	639bp
TuRpt3	RNAi	[T7]-CCTTCAGCTAGTGTCGCCTT	[T7]-CTGGACGGAGTAAAGCAGGG	598bp
Control (NC)	RNAi	[T7]-GCCCTCTCCTGGTTGTAAACTT	[T7]-CGACCCCATCAGGCTATTGA	382bp
TuCOPB2	RNAi	[T7]-AGTTTGTGGTGACGGAGAAT	[T7]-TTTTCCTTCGGCATGTATCC	612bp
TuVATPase	RNAi	[T7]-TCCAACAGTGATGTTATTGTTTACG	[T7]-GAAGAGGTACGAAATCTGGG	628bp
TuCOPB2_100	Localisation	[T7]-AGTTTGTGGTGACGGAGAAT	[T7]-TGAGTCTAGGGCCCAAACAA	97bp
TuCOPB2_400	Localisation	[T7]-AGTTTGTGGTGACGGAGAAT	[T7]-GCTTTGGCAACGTTATCAGG	390bp
**T7 promoter**		TAATACGACTCACTATAGGG		
	**RT-qPCR**			**Amplification efficiency**
TuSrp54	RT-qPCR	TGGTCATGCTTGTGAAGCTC	ACCACCACCTTTAGCGTGTC	97%
TuRop	RT-qPCR	GCCTGCAGATAAGTGCATCA	TTTGCGCTGAATTTGGTACA	91%
TuSnap α	RT-qPCR	TCAGGCGATAGTGAAGCAAA	CTCTAGAAGAGCCCCCGAAC	93%
TuHsc70-3	RT-qPCR	CCTTCGCTCCAGAAGAGATTAG	GGTGACGACAGCATGAGTAA	101 %
TuRpn7	RT-qPCR	TGTTGCAGGACGCAATTAAG	ACGCTCCAGCAGTTCTTTGT	98 %
TuRpt3	RT-qPCR	TGAAAAAGCTACAACGGCAAT	TGTCCAATAACCAAGGGAACA	98%
RP49 (tetur18g03590)	RT-qPCR	CTTCAAGCGGCATCAGAGC	CGCATCTGACCCTTGAACTTC	102 %
CycA (tetur01g12670)	RT-qPCR	GCTTCAAGGCGGTGACTTT	ACCTGGTCCAGTGTGTTTGAG	102%
tetur35g00760	RT-qPCR	GGAGGGGATTCAGGAGAAGG	GCATCAGGACCCTTGCCTTT	101%
tetur02g06210	RT-qPCR	ACAATGTGCTTGGTTTCGCTC	ATTCAGCAACTCTACCGCCT	93%
	***In situ* hybridization**			**Probe size**
TuCOPB2	ISH_Sense	[T7]-GTGAGGTTCCTGTTCGGTGT	TTCAATTCTTCCGGATCGAC	577bp
	ISH_Anti sense	GTGAGGTTCCTGTTCGGTGT	[T7]- TTCAATTCTTCCGGATCGAC	
TuSrp54	ISH_Sense	[T7]-AGCAGTTACCAACTCTCCCA	[AGCAGCCCCCTGTAATTGTC	685bp
	ISH_Anti sense	[AGCAGTTACCAACTCTCCCA	[T7]-AGCAGCCCCCTGTAATTGTC	
TuRop	ISH_Sense	[T7]-CGAGGCTCCAGAAAAACAC	GATGCACTTATCTGCAGGCG	658bp
	ISH_Anti sense	CGAGGCTCCAGAAAAACAC	[T7]-GATGCACTTATCTGCAGGCG	
TuSnapα	ISH_Sense	[T7]-GATTGTTCGGGGGCTCTTCT	TCCCGGGAATCACTGAAAGC	631bp
	ISH_Anti sense	GATTGTTCGGGGGCTCTTCT	[T7]-TCCCGGGAATCACTGAAAGC	
TuHsc70-3	ISH_Sense	[T7]-AACCTACAGCTGCTGCCATT	GGGTTAATGCCCCGAGTAGG	568bp
	ISH_Anti sense	AACCTACAGCTGCTGCCATT	[T7]-GGGTTAATGCCCCGAGTAGG	
TuRpn7	ISH_Sense	[T7]-AACTGCTGGAGCGTATGAGG	AATCTTCGACCCACGCAAGT	639bp
	ISH_Anti sense	AACTGCTGGAGCGTATGAGG	[T7]-AATCTTCGACCCACGCAAGT	
TuRpt3	ISH_Sense	[T7]-CCTTCAGCTAGTGTCGCCTT	CTGGACGGAGTAAAGCAGGG	598bp
	ISH_Anti sense	CCTTCAGCTAGTGTCGCCTT	[T7]-CTGGACGGAGTAAAGCAGGG	
TuVATPase	ISH_Sense	[T7]-CCGTGATATGGGTTACCATG	GAAGAGGTACGAAATCTGGG	416bp
	ISH_Anti sense	CCGTGATATGGGTTACCATG	[T7]-GAAGAGGTACGAAATCTGGG	

Supplemental Table 3. Expression patterns of RNAi-targeted genes

	T. urticae		*T. castaneum*		*D. melanogaster*	
**gene name**	**ID**	**Expression pattern (ISH)**	**ID**	**Transcriptomic profiling (RNAseq, enrichment >1 compared to whole beetle)**	**ID**	**Expression pattern (ISH)**
TuRop	*tetur13g00570*	ovaries, nervous mass	TC011120	ubiquitous; enriched in brain; head; midgut; gonads; tubule; rectal complex; carcass	CG15811	central nervous system (embryonic stage; Salzberg et al., 1993)<br>ubiquitous; larval midgut (embryonic stage; Fisher et al., 2012)
TuSnap a	*tetur06g05400*	ovaries, nurse cells, DC	TC013571	ubiquitous; enriched in brain; midgut; head; tubule; rectal complex; carcass; fat body; gonads	CG6625	larval nervous system (embryonic stage; Fisher et al., 2012; Ordway et al., 1994)
TuRpt3	*tetur32g01800*	ovaries; caeca midgut epithelical	TC007999	ubiquitous; enriched in gonads; fat body	CG16916	N/A
TuRpn7	*tetur33g01390*	ovaries, caeca midgut epithelial	TC006375	ubiquitous; enriched in midgut; carcass; gonads; tubule; fat body; rectal complex; brain; head	CG5378	ubiquitous (embryonic stage; Fisher et al., 2012)
TuHsc70-3	*tetur08g01320*	ovaries, caeca midgut epithelial, epithelium	TC004425	ubiquitous; enriched in midgut; gonads; carcass; fat body	CG4147	N/A
TuSrp54	*tetur17g03110*	ovaries, adjacent nurse cells	TC002574	ubiquitous; enriched in midgut; gonads; fat body	CG4659	salivary gland body primordium;presumptive embryonic salivary gland (embryonic stage; Abrams and Andrew, 2005)
TuCOPB2	*tetur24g00150*	ovaries, caeca midgut epithelial	TC013867	ubiquitous; enriched in midgut; tubule; rectal complex; gonads; brain	CG6699	ubiquitous; midgut; salivary gland; embryonic dorsal epidermis (embryonic stage; Fisher et al., 2012)
TuVATPase	*tetur09g04140*	ovaries; posterior midgut; caeca midgut epithelial; digestive cells	TC008354	ubiquitous; enriched in midgut; rectal complex; brain; tubule; carcass; gonads	CG3762	ubiquitous; midgut; Malpighian tubule; gastric caecum; muscles (embryonic stage; Fisher et al., 2012)<br>Malpigian tubule; ovariole (adult stage; Allan et al., 2005)

A

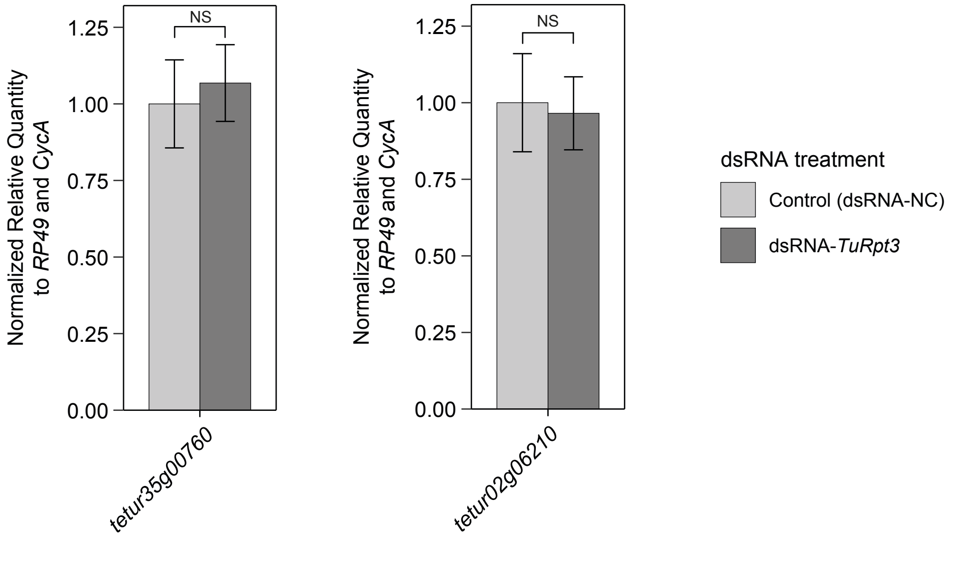

B Pairwise sequence alignment between *tetur35g00760* CDS and dsRNA-*TuRpt3*

```
dsRNA_TuRpt3      1 -----CCTTC-------AGCTA-------GTGTC-------GCCTT----     20
                         .||||       ||..|       |||||       .||||
tetur35g00760   401 TATCAACTTCTGTGGGAAGTGAACATTATGTGTCTATATTATCCTTTGTG    450

dsRNA_TuRpt3     21 -----------ACATAAGCACAGTAATGC----TTTGGTTGA--------     47
                               ||.|.|||.||  |.|||    |||.|||||
tetur35g00760   451 GACAAGGAACAACTTGAGCCCA--ATTGCTCAGTTTTGTTGAATCATAAA    498

dsRNA_TuRpt3     48 --------TGTTTTACCA--CCTGA-AGCTGACTC---TTCTATCG-CTA     82
                            ||||.||..|  |||.| .|.|||..|   |.|.||.| |.|
tetur35g00760   499 GTCCATGCTGTTGTAGGAGTCCTCAGTGATGATACAGATCCAATGGTCAA    548

dsRNA_TuRpt3     83 TGT--TGAGAGCTGATGAAAAA---CCTGATGTTAGTTATGCAGATATTG    127
                    |||  ||| ||.|  .||||||   |||.|.|..|.||||||.|||||||
tetur35g00760   549 TGTAATGA-AGTT--AGAAAAAGCTCCTCAAGAAACTTATGCTGATATTG    595

dsRNA_TuRpt3    128 GTGGTCTCGA-CATTCAAAAGCAAGAAATTCGTGAAGCAGTAGAGCTTCC    176
                    |.|||||||| || .||||..|||||||||...|||.|||||||||||||
tetur35g00760   596 GAGGTCTCGATCA-GCAAATTCAAGAAATTAAAGAATCAGTAGAGCTTCC    644

dsRNA_TuRpt3    177 ATTAACTCACTTTGAATTGTATAAACAAATCGGTATTGACCCACCTCGTG    226
                    .||.|||||....||||..|||.|..||||.||||||.|.||||||...|
tetur35g00760   645 TTTGACTCATCCAGAATACTATGAGGAAATGGGTATTAAACCACCTAAAG    694

dsRNA_TuRpt3    227 GTGTTCTCATGTATGGTCCTCCAGGTTGTGGTAAAACTATGTTAGCCAAA    276
                    ||||..|..|.|||||.||.||.|||...||||||||..|.|||||.|||
tetur35g00760   695 GTGTCATTCTTTATGGCCCACCTGGTACCGGTAAAACACTTTTAGCTAAA    744

dsRNA_TuRpt3    277 GCAGTTGCTCATCATACCACTGCATCTTTCATTCGTGTTGTTGGTTCTGA    326
                    ||.||.|||.||||.||..|.|||.|.|||.|..|.||.|||||.||.||
tetur35g00760   745 GCTGTGGCTAATCAAACATCAGCAACCTTCTTGAGAGTAGTTGGGTCCGA    794

dsRNA_TuRpt3    327 GTTTGTCCAAAAGTACCTTGGTGAAGGTCCTAGGATGGTCCGAGATGTGT    376
                    |||..|.|||||.||.||.||.||.||.||.|...||||..||||..|.|
tetur35g00760   795 GTTGATTCAAAAATATCTCGGAGATGGACCAAAATTGGTTAGAGAATTAT    844
```

```
dsRNA_TuRpt3     377 TTAGACTAGCGCGAGAAAATGCCCCGGCAATTATATTTATTGATGAAATC    426
                     |||||.||||...||||.|.||.|||.|.||..|.|||||||||||||||
tetur35g00760    845 TTAGAGTAGCTGAAGAACACGCACCGTCCATCGTTTTTATTGATGAAATC    894

dsRNA_TuRpt3     427 GATGCTATAGCAACCAAGAGATTTGATGCTCAAA--CTGGTGCTGACAGA    474
                     ||||||||.|..||.||..|||.||||  |||||  ||||.|..||.||.
tetur35g00760    895 GATGCTATTGGTACTAAACGATATGAT--TCAAATTCTGGAGGAGAAAGG    942

dsRNA_TuRpt3     475 GAGGTTCAAAGAATCTTGTTAGAATTATTGAATCAAATGGATGGGTTTGA    524
                     ||..|.|||||||.|.||||.||.|||.|.||.|||.|||||||.|||||
tetur35g00760    943 GAAATCCAAAGAACCATGTTGGAGTTACTTAACCAATTGGATGGTTTTGA    992

dsRNA_TuRpt3     525 TCAAAGTACTAATGTTAAAGTAATCATGGCCACT-----AATCGGGCAGA    569
                     |....||..|.||||.|||||..|.|||||.|||     |||||   |.|
tetur35g00760    993 TTCTCGTGGTGATGTCAAAGTTGTGATGGCAACTAATAGAATCG---AAA   1039

dsRNA_TuRpt3     570 TACTTTGGACCCTG-CTTTACTCCGTCCAG--------------------    598
                     .||||  ||.|||| |.||| |.||.||.|
tetur35g00760   1040 CACTT--GATCCTGCCCTTA-TTCGGCCTGGTCGTATCGATCGTAAAATT   1086
```

C, Pairwise sequence alignment between *tetur02g06210* CDS and dsRNA-*TuRpt3*

```
dsRNA_TuRpt3       1 -----------------------------CC----TTC--------AGCT      9
                                                  ||    |||        ||.|
tetur02g06210    701 ATCTGGGAATAAGCTCCGAAAATGGTGTTCCTGTTTTCTATGAAAGAGGT    750

dsRNA_TuRpt3      10 AGTGTCGCCTTACATAAGC-ACAGTAATGCTTTGGTTG------ATGT--     50
                     .|||       |||..||| ||| |.|..||.|.||||      ||||
tetur02g06210    751 GGTG-------ACAATAGCTACA-TCACTCTGTTGTTGTTAATTATGTTA    792

dsRNA_TuRpt3      51 -----------------TTTACCAC-------CTGAAGCTGAC---TCTT     73
                                      ||||.|||       .|.||| ||.|   ||||
tetur02g06210    793 GCCTTATACGCTCTCTCTTTAACACAAGTCCGATCAAG-TGTCTCATCTT    841

dsRNA_TuRpt3      74 CTATCGCTATGTT--------------GAGAGCTGATGAAAAAC------    103
                     |||  |..|||||              .|||||         ||
tetur02g06210    842 CTA--GTAATGTTTTCTCCAAACTTGGAAGAGC---------ACGTTTCA    880

dsRNA_TuRpt3     104 CTGATGTT--------------------------AGTTA----TGCAGAT    123
                     |||.||||                          |||||    |||.|||
tetur02g06210    881 CTGTTGTTGATTCATTAACAGGGGCAGGTAAAGGAGTTAAATTTGCCGAT    930

dsRNA_TuRpt3     124 ATTGGTGGTCTCGACATTCAAAAGCAAGAAATTCGTGAAGCAGTA--GAG    171
                     .|||..||||||.|     |.|||||| ||||   ||||  |.||  |||
tetur02g06210    931 GTTGCAGGTCTCAA-----AGAAGCAA-AAAT---TGAA--ATTATGGAG    969

dsRNA_TuRpt3     172 CT--TCCATTAACTCA---CT-TTGAATTGTATAAA--CAAATCGGTATT    213
                     .|  ||.||||.||||   || .||||      |||  |||   ||.|||
tetur02g06210    970 TTTGTCGATTATCTCAAAGCTCCTGAA------AAATTCAA---GGCATT   1010

dsRNA_TuRpt3     214 --GACC-----CACCTCGTGGTGTTCT-CATGTATGGTCCTCCAGGTTGT    255
                       ||.|     .||||||.||||||.| |.|.|| ||||||||.||||||
tetur02g06210   1011 AGGAGCTAAAGTACCTCGAGGTGTTTTGCTTTTA-GGTCCTCCTGGTTGT   1059

dsRNA_TuRpt3     256 GGTAAAACTATGTTAGCCAAAGCAGTTG-CTCATCATACCACTGC--ATC    302
                     |||||||||||||||||||||||||||| ||||..|       ||  ||.
tetur02g06210   1060 GGTAAAACTATGTTAGCCAAAGCAGTTGCCTCAGAA-------GCAAATG   1102

dsRNA_TuRpt3     303 TTTCATTCGTG----TTGTTGGTTCTGAGTTTGTCCAAAAGTACCTTGGT    348
                     ||.|.||..||    |.|.|||..|||||||..||.|||.|   .|||||
tetur02g06210   1103 TTCCGTTTTTGGCGATGGCTGGAACTGAGTTCATCGAAATG---ATTGGT   1149
```

```
dsRNA_TuRpt3       349 GAAGGTCCTAGGATG--------GTCCGAGATGTGTTTAGACTAGCGCGA    390
                       |.|    ||.||| |        ||..|||||.|.||.|.|..|||.|||
tetur02g06210     1150 GGA----CTTGGA-GCATCTAGAGTGAGAGATCTTTTCAAAGAAGCTCGA   1194

dsRNA_TuRpt3       391 GAAAATGCCCCGGCAATTATA-----TTTATTGATGAAATCGATGCTAT-    434
                       .|||..||.||     ||.||     |.||||||.|||||||||||.||
tetur02g06210     1195 AAAAGAGCACC-----TTGTATAGTTTATATTGACGAAATCGATGCAATC   1239

dsRNA_TuRpt3       435 ---AGCAACCAAG----------AGATTTGATGCT---------------    456
                          ||.||  |||          |||.|.||..||
tetur02g06210     1240 GGAAGGAA--AAGGAGTGGAACTAGAGTCGAAACTTCAGGAGAAGAAGAA   1287

dsRNA_TuRpt3       457 CAAAC---------------------------TGGT---GCTG-------    469
                       |||||                           ||||   ||||
tetur02g06210     1288 CAAACTCTAAATCAATTGCTAGTTGAAATGGATGGTATGGCTGGCCGAGA   1337

dsRNA_TuRpt3       470 --------------------------ACAGA---GAGGTTC---------    481
                                                 |||||   ||.||||
tetur02g06210     1338 AGGTGTCATTCTTTTAGGGTCGACAAACAGAGCTGAAGTTCTGGACAAAG   1387

dsRNA_TuRpt3       482 --------------------------------------------------    481

tetur02g06210     1388 CACTACTTCGACCTGGTAGATTTGATCGACATATTCTCATAGATTTACCT   1437

dsRNA_TuRpt3       482 ---------------AAAGAATCTTGTTAGAATTA-TTGAATCAAATGGA    515
                                      ||||||.|||.|.||||.|| |||||  ||||..|
tetur02g06210     1438 ACACTAGAAGAAAGAAAAGAAACTTTTGAGAAATACTTGAA--AAATATA   1485

dsRNA_TuRpt3       516 TGGGTTT-GATCAA---AGTACTAATGTTAAAGTAATCA----TGGCCAC    557
                       ....||| |||.||   ||.|  |||||    .|.||||    |||||.|
tetur02g06210     1486 AAACTTTCGATAAAACCAGAA--AATGT----CTCATCACGTCTGGCCTC   1529

dsRNA_TuRpt3       558 T---AATCGGGCAGATACTTTGGACCC-----------TG----------    583
                       |   |.||..|  |||.|..||||.||           ||
tetur02g06210     1530 TTTAACTCCTG--GATTCAGTGGAGCCGATATCGCCAATGTGTGCAATGA   1577

dsRNA_TuRpt3       584 ------CTTTACTCCGTCCAG-----------------------------    598
                             |.|||.|.||.|.||
tetur02g06210     1578 ATCAGCCCTTAATGCGGCTAGATATAAACGAAAAGCAGTTACTCCTAATG   1627
```

Supplemental Figure 1. Expression analysis and pairwise sequence alignments of genes with consecutive 21 bp-sequence identity to dsRNA-*TuRpt3*. **A,** Expression analysis of *tetur35g00760* and *tetur02g06210* displaying consecutive 21 bp-sequence identity to dsRNA-*TuRpt3*. Average gene expression level relative to the expression of the reference control genes *RP49* and *CycA*. Data represent the mean ± SE. The RT-qPCR analysis was conducted in three independent experimental runs. Statistical analysis was performed using unpaired two-tailed t-test (exact P-values corresponding to each pairwise comparison between the control and the treatment are displayed; NS: not significant). **B,** Pairwise sequence alignment between *tetur35g00760* CDS and *TuRpt3* cDNA sequence used to generate dsRNA-*TuRpt3*. EMBOSS Needle was used to perform the alignment. Consecutive 21 bp-sequence identity to dsRNA-*TuRpt3* is displayed in yellow. **C,** Pairwise sequence alignment between *tetur35g00760* CDS and *TuRpt3* cDNA sequence used to generate dsRNA-*TuRpt3*. EMBOSS Needle was used to perform the alignment. Consecutive 21 bp-sequence identity to dsRNA-*TuRpt3* is displayed in yellow.
